# Removal of an Impaled Intraocular Hair Comb Following Self-inflicted Trauma

**DOI:** 10.5811/cpcem.2019.10.44460

**Published:** 2020-01-02

**Authors:** Michele Markovitz, Jordan Hamburger, Brian S. Fromm, Brendan Carr, Xiao Chi Zhang

**Affiliations:** * Wills Eye Hospital, Department of Ophthalmology, Philadelphia, Pennsylvania; † Sidney Kimmel Medical School, Philadelphia, Pennsylvania; ‡ Thomas Jefferson University, Department of Emergency Medicine, Philadelphia, Pennsylvania

## Abstract

Ocular trauma is one of the most common and vision-threatening ophthalmic presentations with a wide spectrum of complications, such as bleeding, infection, vision loss, and enucleation. A 64-year-old-male presented to the emergency department (ED) with a self-inflicted orbital penetrating injury with a hair comb. Computed tomography showed the comb traversed the medial orbit inferior to the medial rectus but did not damage the optic nerve; there were no globe or orbital wall fractures. His ocular exam was significant for a right eye afferent pupillary defect and decreased visual acuity 20/800, consistent with optic neuropathy. Primary concerns were stabilizing and removing the foreign body without causing further damage in the setting of an uncooperative patient. The comb was removed with the aid of local and systemic analgesia using gentle traction and normal saline irrigation. The patient was admitted for systemic and topical antibiotics and showed improvement in visual acuity and resolution of his optic neuropathy. This case illustrates the importance of rapid ED assessment and management of complex penetrating ocular trauma. Examination should specifically look for signs of globe rupture and optic nerve injury. Expedited foreign body removal should be managed together with an ophthalmologist with procedural sedation and broad-spectrum antibiotics to avoid further visual and infectious complications.

## INTRODUCTION

Ocular trauma is one of the most common, vision-threatening presentations seen by emergency physicians (EP).^1^,^2^ Complications include bleeding, infection, vision loss, loss of the eye and, rarely, sympathetic ophthalmia.^1^ Emergency management strategies differ depending on the type, size, and site of penetration; however, timely imaging, adequate sedation, proper antibiotic, and early anti-inflammatory treatment can reduce morbidity and permanent vision loss.

## CASE REPORT

A 64-year-old-male with a medical history of alcohol dependence with end-stage liver disease, and unknown baseline visual acuity, presented to the emergency department (ED) for a foreign body (FB) in his right eye. The patient was receiving treatment at an outside hospital for hepatic encephalopathy when he forcibly impaled the handle of a plastic comb into his right eye without provocation. The patient was transferred to our ED due to its affiliation with an independent eye hospital and for complex ophthalmic intervention. Prior to arrival, the patient had received intravenous (IV) fentanyl for pain control and agitation, undergone a computed tomography (CT), and was immediately transferred with the comb secured in place with bulky dressing.

**Image 1:**
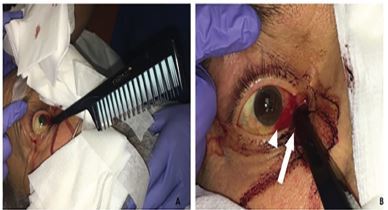
Hair comb impaled into the right inferomedial orbit (A) with higher magnification (B) of the foreign body with right conjunctival laceration at the nasal limbus extending inferonasally (arrow) with associated subconjunctival hemorrhage (arrowhead).

On arrival, the patient was sedated, jaundiced, and intermittently following simple commands. His vital signs were blood pressure 138/66 millimeters of mercury (mmHg), pulse rate 68 beats per minute, temperature 98^o^ Fahrenheit, and saturating 97% on room air. External examination revealed an intact plastic hair comb handle embedded into the inferomedial right orbit with a right conjunctival laceration at the nasal limbus extending inferonasally with associated subconjunctival hemorrhage (Image 1).

**Image 2:**
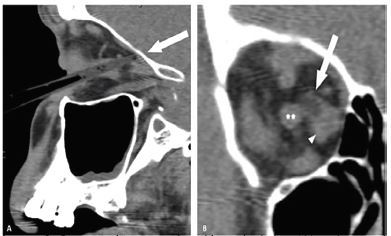
Computed tomography with sagittal view (A) and coronal view (B) showing the foreign body (arrow) entering the orbit, adjacent to the right medial rectus muscle (arrowhead) and abutting the optic nerve sheath (white asterisks in middle of figure) and terminating at the posterior superior orbital wall with no evidence of orbital wall fracture.

Ophthalmology was consulted immediately on patient arrival and was at bedside within 30 minutes. While all aspects of the eye exam were limited by cooperation and sedation, the patient was able to perceive light in both eyes and cooperated enough to determine a visual acuity in the right eye of 20/800 and a full left eye extraocular movement. Pupillary exam showed equally round and reactive pupils with a 1+ afferent pupillary defect (APD) in the right eye. Despite his encephalopathy, the patient was cooperative and directable and allowed for rapid intraocular pressures (IOP) measurement using gentle eyelid traction and tonopen tonometry with IOP measuring 13 mmHg and 10 mmHg (reference range 8–21 mm Hg). The CT from the transferring hospital demonstrated the comb was localized in the medial orbit adjacent to the medial rectus, extending toward the orbital apex and abutting the optic nerve, but it did not penetrate through the orbital wall and the globe was intact (Image 2).

To avoid further ocular damage, the decision was made to emergently remove the comb at the bedside with topical proparacaine and IV fentanyl. The ophthalmologist gently removed the comb using steady traction after sterilizing the surgical field with topical betadine. The patient was admitted for further management of his hepatic disease and psychiatric evaluation. He was continued on topical erythromycin ointment and neomycin-polymyxin-dexamethasone drops, IV piperacillin/tazobactam, and IV levofloxacin. He also received three days of IV dexamethasone for optic neuropathy. His afferent pupillary defect resolved by hospital day 2 and his visual acuity improved to 20/70 in both eyes. On discharge (hospital day 13), he had regained full extraocular motility, and his conjunctival laceration and subconjunctival hemorrhage were healing well and did not require further procedural intervention. He was discharged with erythromycin ointment and neomycin-polymyxin-dexamethasone drops.

**CPC-EM Capsule**

What do we already know about this clinical entity?

Intraorbital penetrating trauma can lead to infection, vision loss, and many other medical problems.

What makes this presentation of disease reportable?

This is a novel presentation of rare disease in which a penetrating intraorbital foreign body (FB) resulted in vision impairment without globe damage.

What is the major learning point?

Unstable, penetrating intraorbital FB without globe rupture or intracranial extension may be removed in the ED with appropriate imaging, sedation, and ophthalmology consultation.

How might this improve emergency medicine practice?

This case highlights the sedatives, antibiotics, anti-inflammatories, and procedural techniques needed in the removal of foreign bodies in unpredictable patients.

## DISCUSSION

Penetrating ocular trauma is a main cause of unilateral blindness.^3^ Management strategies depend on the type, size of the FB, site of penetration, and hemostasis.^4^–^7^ Ocular imaging of radiolucent FBs remains difficult: plain films detect only metallic FBs^8^ and magnetic resonance imaging poses risk associated with unrecognized metal.^9^ Inert FBs carry a lower risk of infection than organic FBs such as wood,^4^ but still warrant broad-spectrum antibiotics to prevent infections.^10^,^11^

Intraorbital trauma should be explored for globe rupture and optic nerve injuries.^12^–^14^ Classic signs of optic nerve compromise include decreased visual acuity, APD, and dyschromatopsia.^15^ In our patient the imaging demonstrated an intact globe and the presence of a transient optic neuropathy, as evidenced by initial presence of APD, likely attributable to perineural inflammation from the adjacent FB rather than an irreversible traumatic optic neuropathy due improvement after FB removal.

In many cases, delayed removal of the FB has shown no negative impact on the final visual outcome.^16^,^17^ Some intraorbital FBs such as glass, plastic, graphite, aluminum, and gold can be left in the eye if they pose no danger to intraocular structures, while iron, lead, and copper necessitate immediate removal due to retinal toxicity, chalcosis, and siderosis risks.^18^ Immediate removal of an intraorbital FB may result in incomplete retrieval or fragmented pieces left in the orbit, necessitating immediate transfer to a tertiary center with ophthalmology for emergent globe exploration. Further potential complications include bleeding and compartment syndrome, requiring surgical decompression with lateral canthotomy and cantholysis.

In our case, there was a high concern that instability of the comb and patient agitation could dislodge it and cause permanent optic nerve damage or even penetration into the intracranial cavity. Procedural sedation with medications such as ketamine or propofol may be considered for reducing agitation and preventing further intraocular injuries.^19^ While ketamine may be associated with increased IOP, our patient did not demonstrate ocular compartment syndrome or elevated IOP that would have limited the use of this chemical sedative for a time-sensitive and vision-saving procedure. Ophthalmic anesthetic drops should also be used for removal of any FB involving the cornea or conjunctiva; the need for additional medications should be at the discretion of the treating physician based on the individual situation. Fortunately, the FB was removed with limited analgesia and his APD improved shortly after FB removal and systemic steroids.

## CONCLUSION

This case highlighted the risks and benefits in removing an unstable, intraorbital foreign body in an unpredictable patient in a time-sensitive and vision-threatening situation. Examination should assess for globe rupture and optic nerve injury. CT imaging can guide both prognosis and management. Early ED management should include aggressive pain control, careful chemical sedation and early broad-spectrum antibiotics to avoid further traumatic, visual, and infectious complications. Cases requiring expedited foreign body removal should be managed together with an ophthalmologist.
